# What is the role of cheminformatics in a pandemic?

**DOI:** 10.1186/s13321-021-00491-6

**Published:** 2021-03-02

**Authors:** Rajarshi Guha, Egon Willighagen, Barbara Zdrazil, Nina Jeliazkova

**Affiliations:** 1 Vertex Pharmaceuticals, 50 Northern Ave, Boston, MA 02210 USA; 2 Maastricht University, Universiteitssingel 50, 6229 ER Maastricht, Netherlands; 3 University of Vienna, Althanstraße 14, 1090 Vienna, Austria; 4 Ideaconsult Ltd, 1000 Sofia, Bulgaria

**Keywords:** Cheminformatics, COVID-19, Virtual screening, Pandemic, SARS-CoV-2

The COVID-19 pandemic has led to a spike in research output [1, 2] surrounding all aspects of the disease, ranging in scale from the molecular to the population level. There have been many preprints (and subsequent journal publications) in the field of cheminformatics that attempt to address the discovery of therapeutics against the disease. For example, numerous virtual screening publications have proposed potentially interesting candidates (for an overview see this Scholia page [3]). During a recent conversation with our Editorial Board we discussed the possibility of a thematic issue in the Journal of Cheminformatics on COVID-19. We have decided instead to maintain our focus on novel cheminformatics and reiterate the requirement that studies proposing compounds as putative prophylactics or therapeutics be backed up by experimental validation, irrespective of whether the target is COVID-19 or some other disease.

## Computation is necessary, but not sufficient

The urgency of the COVID-19 epidemic presents a number of challenges for the computational chemistry and informatics research community. While control and mitigation of viral spread is a primary focus of health systems, this is closely tied to identifying pre-existing or novel therapeutic approaches to treat the disease itself.

Cheminformatics approaches are one set of tools in the computational toolbox that can be applied to therapeutic discovery. Given the availability of Open Source and commercial tools, coupled with public data, we have seen many studies that have prioritized compounds as potential therapeutic candidates. From the point of view of this journal, straightforward applications of pre-existing or well-known pipelines are out of scope for research articles [4].

However, one might argue that in such a crisis situation, dissemination of all such applications could be beneficial. Indeed, while more knowledge is useful in the current pandemic, we believe that it needs to be rigorous knowledge. A particularly egrigeous example is applications of drug repurposing pipelines. Given the current state of the art it is very easy to propose lists of approved or investigational drugs, that *could* serve as COVID-19 therapeutics. While it is possible to make justifications for some of these based on prior knowledge of mode of action, it still remains that these are *hypotheses*. In our opinion, the urgency of the current pandemic requires that predictions be validated by experiment, and we can no longer carry on (computational) business as usual.

While testable hypotheses are a key requirement in the current setting, it is equally important that the pipeline used to reach such hypotheses be as rigorous as possible. For statistical and machine learning based approaches, appropriate statistical methodology should be employed [5–7]. Similarly, best practices should be followed for ligand-based [8, 9] and structure-based approaches [10, 11]. While we expect all work submitted to the journal to adhere to these practices, these aspects become even more important when computational work with experimental components are submitted to non-computational journals.

## Open is a starting point

The challenge of the current pandemic is to identify novel therapeutics in a rapid but rigorous fashion. This suggests that novel method development may not be well suited to the current scenario. On the other hand, data being generated, either experimentally or computationally can serve as a foundation for computational studies. This is enabled by ensuring such data is made available in an open fashion and following FAIR principles [12, 13]. For example, see the COVID-19 Wikiproject [14] that aims to make drug discovery related data FAIR. Yet, it is important to remember that even when data and methods are shared openly, they may not actually be effective, as in a crisis situation, such as the COVID-19 pandemic, researchers will tend to stick to what they know. But more importantly, they will tend to stick to what they know works. We believe that in such a scenario, methodology development takes a back seat to data publication, and ensuring that the relevant data is made available and findable efficiently is a key task.

## Cheminformatics in action

It is important to note that there are examples of work that exemplify computational-experimental collaboration and the community response to disseminating the plethora of computational studies and their data. We highlight two of them, namely the COVID Moonshot [15] and the COVID-19 Molecular Structure and Therapeutics Hub [16]. The COVID Moonshot focuses on finding inhibitors of the Main protease (Mpro) of COVID-19, and involves 300 participants with a core group of 20 people. Importantly, the project has access to computational, synthetic and bioassay resources, coupled to a synchrotron that produces multiple structures each week. The project has been able to identify Mpro binders exhibiting nanomolar potencies. While the project employs well-known computational methodologies, the key element is that computational results are part of the design-make-test cycle. In other words, computation is not isolated. Nonetheless, the project makes use of key Open Source products such as Fragalysis (a cloud-based application to progress hits in fragment-based drug design projects) [17]. The Hub, on the other hand, is an example of a resource that aggregates data of various types that can be used for computational studies. This includes structure models, simulation related datasets (e.g., configuration files, trajectories). While this does not directly lead to tested small molecules, it represents an invaluable coordinated resource covering the wide variety of computational studies that are being published.

Given that rapid dissemination via preprints and resources such as the Hub are more appropriate for the immediate response required in the current setting, it is reasonable to ask how effective these resources are in covering the research landscape and driving COVID-19 research in the computational chemistry domain. Such an evaluation is probably better suited for a more in-depth study, but we note that efforts such as the COVID Moonshot and the Hub now involve contributions from at least 15 industrial and academic groups from across the world.

## Conclusion

It is clear that the computational chemistry and cheminformatics community are actively engaged in COVID-19 research and the rapid appearance of computational research [18] on COVID-19 attests to the value of Open Data, Open Source and Open Science in general. However, given the scope of this journal, and the desire to encourage rigorous cheminformatics studies, we have decided not to create a thematic issue focused on COVID-19, and rather continue to focus on actionable cheminformatics, irrespective of any specific disease. And when computational hypotheses are presented, we continue to require experimental validation.

The above discussion might suggest that the *only* role of cheminformatics is to identify new therapeutic interventions. While this is a key and pressing role in the current pandemic, there are many other areas such as databases (e.g., ChEMBL [19], which underlies a number of virtual screening studies, the NCATS COVID-19 OpenData Portal [20] which rapidly disseminates in vitro screening data against multiple SARS-CoV-2 targets and the canSAR Coronavirus Discovery resource [21] which collates multiple molecular and clinical data types related to COVID-19), literature (e.g., CORD-19 [22] which has collated scholarly articles around SARS-CoV-2 and related coronaviruses to enable text mining research), protein features such as post-translational modifications, force fields, physicochemical properties and associated models that underly many of the approaches that one can use to identify new therapeutics. We would argue that these foundational areas are also critical to ensuring that when the need arises for computational methods to be applied to therapeutic development, they can do so on a solid foundation.

Thus, we hope that cheminformatics researchers will consider the role of chemical information, methods and standards in the context of anti-viral research, and look forward to such submissions. But given the urgency of the current situation, and the need to focus resources on actionable outcomes, we suggest that there are other platforms for the publication of lists of putative inhibitors of a SARS-CoV-2 enzyme. The journal will continue to focus on studies which advance cheminformatics and can be applied both in this pandemic and in the next one.
